# Ethics reporting practices in randomized controlled trials of physical therapy interventions after stroke

**DOI:** 10.1186/s40945-018-0049-9

**Published:** 2018-06-05

**Authors:** Francesco Ferrarello, Matteo Viligiardi, Mauro Di Bari

**Affiliations:** 1 Functional Rehabilitation, Azienda USL Toscana Centro, Via Cavour 118/120, 59100 Prato, Italy; 2 Outpatient Rehabilitation, CRT Clinica di Riabilitazione Toscana Terranuova Bracciolini Spa, Via Gaetano Donizetti 2, 52028 Terranuova Bracciolini, AR Italy; 3 Department of Experimental and Clinical Medicine, Research Unit of Medicine of Aging, University of Florence, and Azienda Ospedaliero–Universitaria Careggi, Viale Pieraccini 18, 50139 Florence, Italy

**Keywords:** Ethics reporting, Physical therapy, Randomized controlled trials, Stroke

## Abstract

**Background:**

Adequate reporting of ethics-related research methods promotes convergence on best ethics practices. In physical therapy, studies on ethics reporting are limited to few aspects, and none focuses on stroke research. Our objectives were to investigate the reporting of multiple ethics-related features and its variation over time, and the characteristics of the studies associated with ethics reporting in Randomized Controlled Trials (RCTs) of physical therapy interventions after stroke.

**Methods:**

A random sample of RCTs published in the years 2004, 2009 and 2014, was extracted from the PubMed database, regardless of the publishing journal. For each trial we investigated year of publication, trial registration, sample size, stroke subtype, phase of the disease, setting, interventions and dosing, outcome measures, outcome of the study, PEDro score and 5-year impact factor of the publishing journal. Reporting of ethics-related issues was analyzed. Differences between groups were examined. Multiple regression was used to evaluate the relationship between ethics-related issues reporting and some studies’ characteristics.

**Results:**

Eighty studies were reviewed. Groups differed in the proportion of registered trials (*p* = .009), 5-year impact factor (*p* = .011), assessment of cognitive capacity (*p* = .049), declaration about conflict of interest (*p* < .001), and number of ethics-related issues reported (*p* = .009). The proportion of issues reported ranged from 92.5% (consent obtaining) to 0% (eventual follow up care). Post-hoc comparisons showed significantly greater reporting of ethics issues in trials published in the year 2014 compared to 2004 (*p* = .014, 95%CI = 0.40/4.26). Year of publication and PEDro score were significant predictors of adequate reporting.

**Conclusions:**

Authors, editors, and reviewers should be more rigorous and demanding about the reporting of ethic-related methods in randomized controlled trials of physical therapy interventions after stroke.

## Background

The importance of ethics in clinical research has been well-established for many decades [1]. A growing attention is given to determine the best ethical practices for conducting observational and experimental studies [2]. Methodological quality, approval by a research ethics committee, and obtaining informed consent from participants are the main ethical issues in research with human beings [3].

Randomized controlled trial (RCT), systematic review and meta-analysis of RCTs are considered reliable research designs, able to evaluate the effectiveness of an intervention [1]. The design of a clinical trial implies adherence to challenging and multifaceted ethical and methodological standards that must integrate each other [4]; Ashton et al. in their taxonomy indeed identified five major categories, with over 5900 possible standards [4]. Differences among research topics and study designs may add further variability in ethical requirements [5–8]. Research conducted with little methodological rigor does not lead to knowledge or benefit, and exposes participants to unnecessary burden or harm [9].

Thus methodological and ethical requirements of a study have a symbiotic relationship [10] and, in order to make readers able to fully evaluate clinical research, both should be appropriately reported [3]. Adequate reporting of research findings may promote the implementation of evidence-based clinical practice in many fields, including physical therapy [11]. However, reporting of scientific methods receives considerably greater attention, as compared with reporting of research ethics issues [12]. Many guidelines have been published to improve the reporting of RCTs, and adherence to these guidelines is usually considered necessary for publication [12]. Recommendations on how ethics issues should be reported in research studies are also available [13], although evidence suggests flaws in publication requirements and reporting of ethical protections [14]. Thus, descriptions provided by journal articles contain little information about research ethics methods [12].

Research reproducibility refers to the possibility to duplicate the results of prior studies, and is based on a clear and comprehensive description of study design [15]. The concept of ethical reproducibility was recently introduced. Ethical reproducibility prescribes thorough reporting and critical evaluation of the ethics methods employed in biomedical research [16]. Although ethics committee approval is a crucial aspect of ethics in research and is usually considered as a proxy measure for the fulfillment of all ethical requirements in research [14], it is not sufficient to judge the overall ethical quality of an RCT [17]. Including ethics-related methods in research reports may address concerns raised by researchers, clinicians, and other stakeholders [2]. Moreover, ethical reproducibility can promote benefits such as learning, inner reflection, increase of ethical responsibility, critical research assessment, and use of better ethical practices [12].

In the physical therapy field, interest on ethical issues of the profession is remarkable, yet it mainly focuses on clinical practice, whereas only few papers have been published over the years addressing ethics in physical therapy research [18]. Studies on issues related to ethics in RCTs reporting were limited to ethics committee approval, informed consent, and confidentiality [14, 19].

Although stroke represents a major topic in physical therapy research [20], none of the studies published on ethical aspects of physical therapy research focused on stroke. Therefore, because available knowledge does not appear to be exhaustive and satisfactory, we designed the present study with the aim to investigate ethics reporting characteristics in RCTs of physical therapy interventions after stroke.

## Methods

The reporting of this study conforms to the Strengthening the Reporting of Observational studies in Epidemiology recommendations [21]. Based on a sample of RCTs of physical therapy interventions after stroke, our objectives were to investigate the reporting of multiple ethics-related features and its variation over time, as well as the characteristics of the studies associated with ethics reporting.

A random sample of RCTs indexed in PubMed was extracted, regardless of the publication journal. To be included, the studies had to be published in English in the years 2004, 2009 and 2014, involve a parallel-group design, and evaluate an experimental physical therapy intervention administered to adult (age ≥ 18 years) individuals with stroke.

The rationale for choosing the three years was the following. The year 2014 had just ended when we drafted the protocol; it was chosen to give a contemporary view. To have a time perspective, we chose the years 2004 and 2009 as comparators, thus covering a period of ten years.

Interventions were considered suitable if included in the classification proposed by De Jong et al. [22], or listed in the book “Guide to Physical Therapist Practice”[23], or reviewed in the Clinician’s Handbooks of the Evidence-Based Review of Stroke Rehabilitation-motor rehabilitation [24].

For the selection of the studies, the term “stroke” followed by “physical therapy” was entered in the search box, filtered by the type of study (RCT). Additional filters related to the year of publication (2004, 2009 and 2014) were then added one at a time to obtain a list of studies for each of the years of interest. The resulting search strategy was the following: ((“stroke”[MeSH Terms] OR “stroke”[All Fields]) AND (“physical therapy modalities”[MeSH Terms] OR (“physical”[All Fields] AND “therapy”[All Fields] AND “modalities”[All Fields]) OR “physical therapy modalities”[All Fields] OR (“physical”[All Fields] AND “therapy”[All Fields]) OR “physical therapy”[All Fields])) AND Randomized Controlled Trial[ptyp] AND ((“2004/01/01”[PDAT]: “2004/12/31”[PDAT]) OR (“2009/01/01”[PDAT]: “2009/12/31”[PDAT]) OR (“2014/01/01”[PDAT]: “2014/12/31”[PDAT])).

**Fig. 1:**
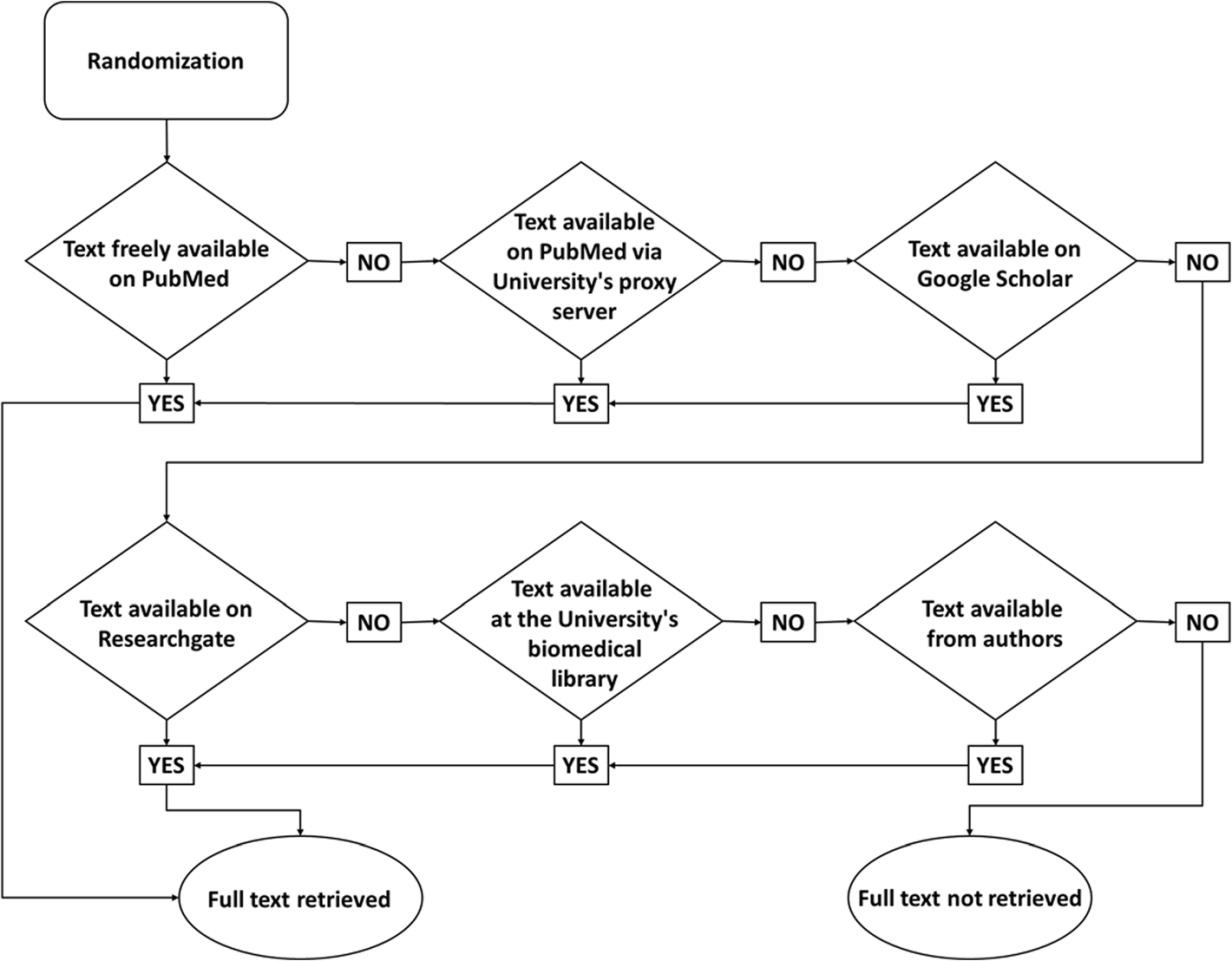
Full text retrieving flow chart

In each year, a random sample of the studies in the PubMed-generated list was extracted. A formal sample size calculation was not performed because of lack of preliminary data; indeed, previous studies differed from the present one in eligibility criteria, sources and methods of studies selection, and variables of interest. In the absence of references, to quantify the sample size we were inspired by Pinto et al. research on RCTs registration,[25] and we established 20 % of the studies as the target sample. We performed calculations based on the total number of articles present in the lists, before the screening process. The random selection was performed in August 2015, by entering the sequence numbers of the lists acquired through search in an on-line randomization program [26]; the studies were then assessed for eligibility following the randomized sequence, until the desired sample size was reached. Full texts of the articles were retrieved following a flow-chart (Fig. 1).

Reasons for exclusion were given for each study excluded. Two reviewers (MV; FF) independently evaluated the studies selected for final inclusion; disagreement was resolved by consensus.

Relevant data were extracted using a standard data recording spreadsheet, including characteristics of the studies and research ethics-related issues.

Data related to year of publication, clinical trial registration, sample size, stroke subtype, phase of the disease, setting, interventions administered, dosing of the interventions, outcome measures, and outcome of the study were extracted from each article included. Information about 5-year impact factor of the publishing journal was acquired [27].

In the absence of a standardized comprehensive list, issues related to ethics in RCTs reporting were chosen considering what has been contemplated in previous publications [2, 14, 17, 28]. The following aspects were investigated in each article:

- ethics review committee or institutional review board study approval
- details about the ethics committees
- obtainment of informed consent
- details about the consent process
- incentives or compensation for participants
- details about incentives or compensation given to participants
- funders
- details about funders
- potential conflicts of interest
- details about conflicts of interest
- steps taken to assess if eligible individuals were able to provide informed consent (e.g. use of validated screening tools for cognitive ability)
- measures taken to ensure the best interests of participants with reduced competence
- steps taken to ensure that the sample size was sufficient to achieve adequate statistical power
- appropriateness of comparators
- justification for the eventual use of placebo
- potential harm for participants
- plans for collecting, assessing, reporting, and managing adverse events and other unintended effects of trial interventions or trial conduct
- appropriate follow up care
- steps taken to prevent unauthorized access to personal and clinical data (confidentiality)
- accordance with the Helsinki Declaration.

We assumed the definition of appropriateness of comparator proposed by Caprino and Russo [29]; in the presence of multiple interventions groups, we considered the most active comparator intervention. For each study included, the number of ethics-related issues reported was calculated by summing up any reported or mentioned issue. The maximum achievable was 22 issues. The extended list of questions is detailed in the Appendix. Data were extracted from an investigator (MV) and subsequently double-checked (FF). Any disagreement was resolved by consensus. The PEDro score of methodological quality was verified for each study [30]. This tool is reliable [31] and useful for assessing the quality of studies in stroke rehabilitation [30]. Criteria for quality assessment are represented by randomness and concealment of allocation, baseline comparability between groups, blinding of participants, therapists, and assessors, adequacy of follow-up assessments, intention-to-treat analysis, between-group comparisons, reporting of point estimates and variability. The PEDro score ranges from 0 (poor quality) to 10 (excellent quality); a score of 6 is conventionally considered as a threshold to identify high-quality studies [32].

### Statistical analysis

Descriptive statistics was initially performed. Five-year impact factor equal to zero were attributed to the three journals not found in the Journal Citation Reports database [27]. In one case where only the impact factor was available, the same value was assigned as 5-year impact factor. Expectation-maximization imputation was used to address missing values (2.5 and 5% respectively) of the number of sessions per week and the minutes per session needed to calculate the dosing of the intervention in hours. Shapiro-Wilk’s test was used to test the normality of distribution. Differences between groups based on the year of publication were examined using the Kruskal-Wallis or the one-way ANOVA tests for interval variables. Pearson χ^2^ test was used for nominal and ordinal variables, except when cell counts were < 5, in which case Fisher exact test was used. Linear trends (across years) were considered as appropriate. In case of significant results, post-hoc tests were performed to explore differences between any two pairs of years. Multiple linear regression was performed to assess the ability of year of publication, trial registration, PEDro score, and 5-year impact factor to predict the number of ethics-related issues reported in each article selected. Preliminary analysis was performed to ensure there was no violation of the assumption of normality, linearity, and multicollinearity.

Analyses were performed using IBM SPSS Statistics for Windows (version 20.0; IBM Corp, Armonk, NY). The significance level was set at a *p* value of <.05.

## Results

**Fig. 2:**
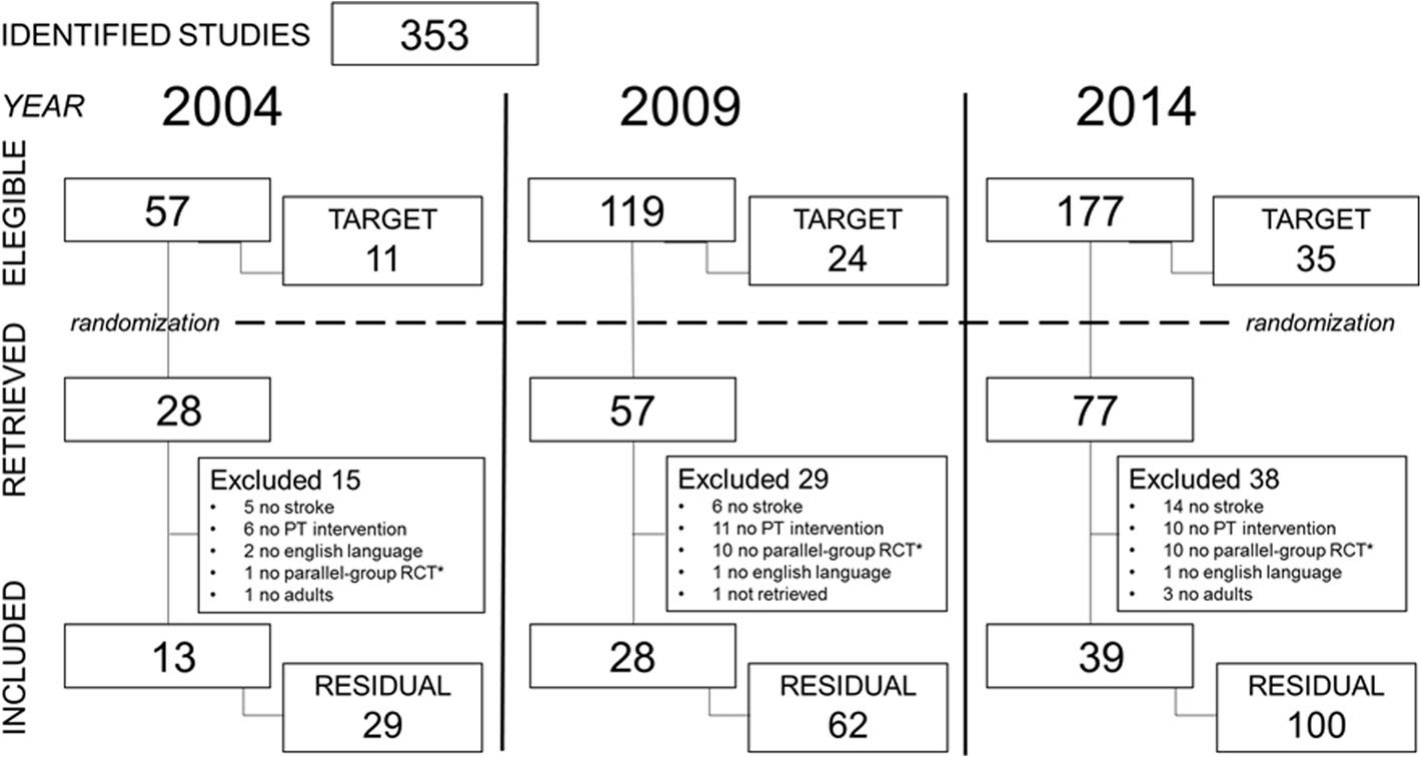
Studies selection flow chart. /*Including protocols, observational studies, preliminary reporting (e.g. recruitment or sample characteristics), non-randomized trials, and within-subject or cross-over design studies. PT = Physical Therapy. RCT = Randomized Controlled Trial

The search on PubMed generated lists of 57, 119, and 177 articles (total 353) for the years 2004, 2009, and 2014, respectively. The target sample size was set at 11 studies for 2004, 24 studies for 2009, and 35 studies for 2014. After randomization, a total of 162 papers were retrieved; the selection process led to the inclusion in the study of 80 articles (Fig. 2).

### Study characteristics

Studies’ characteristics are presented in Table 1. Eighteen percent of the selected trials had been registered. The proportion of registered trials increased substantially across the three years; post-hoc comparison showed that the probability to be not registered was greater for trials published in the years 2004 and 2009 compared to those published in the year 2014 (Table 2).

**Table 1:** Characteristics of the studies

Year	2004 (*n* = 13)	2009 (*n* = 28)	2014 (*n* = 39)	*p*	All studies (*n* = 80)
5-year Impact factor^a^	2.967 (2.447/4.625)	4.206 (2.455/4.626)	2.179 (1.647/3.503)	.011^b^	2.784 (2.130/4.626)
Trial registered^c^	0 (0.0)	2 (7.1)	12 (30.8)	.009^d^	14 (17.5)
Sample size^a^	28 (20.0/47.0)	39 (31.5/65.0)	34 (23.0/52.5)	.212^b^	38 (23.5/56.5)
Intervention groups^c^					
2 groups	11 (84.6)	21 (75.0)	34 (87.2)	.418^d^	66 (82.5)
≥ 3 groups	2 (15.4)	7 (25.0)	5 (12.8)		14 (17.5)
Etiology^c^					
Ischemic	1 (7.7)	8 (28.6)	5 (12.8)		14 (17.5)
Hemorrhagic	0 (0.0)	0 (0.0)	1 (2.6)	.305^d^	1 (1.2)
Mixed	7 (53.8)	16 (57.1)	21 (53.8)		44 (55.0)
None declared	5 (38.5)	4 (14.3)	12 (30.8)		21 (26.2)
Phase of the disease-onset^c^					
< 6 months	4 (30,8)	15 (53.6)	14 (35.9)		33 (41.2)
≥ 6 months	8 (61.5)	12 (42.9)	23 (59.0)	.516^d^	43 (53.8)
None declared	1 (7.7)	1 (3.6)	2 (5.1)		4 (5.0)
Placebo-sham comparator^c^	2 (15.4)	5 (17.9)	9 (23.1)	.807^d^	16 (20.0)
Intervention dosing-hours^ae^	18.0 (6.0/22.5)	15.5 (11.0/27.0)	24.0 (10.0/34.7)	.139^b^	18.0 (9.0/30.0)
At least one outcome^c^					
Favorable	9 (69.2)	20 (71.4)	25 (64.1)	.855^d^	54 (67.5)
Nonsignificant	11 (84.6)	25 (89.3)	36 (92.3)	.614^d^	72 (90.0)
Unfavorable	1 (7.7)	1 (3.6)	3 (7.7)	.705^d^	5 (6.2)
PEDro score^a^	6 (5.0/6.0)	6 (5.0/7.0)	6 (5.0/7.5)	.073^b^	6 (5.0/7.0)

^a^ median (1th quartile/3rd quartile)

^b^ Kruskal-Wallis test

^c^ absolute frequencies (percentages)

^d^ Fisher exact test

^e^ after Expectation-maximization imputation

**Table 2:** Post-hoc test results

Variable		95% CI	*p*
Trial registration^a^			
2004 vs 2009	1.077	.972 to 1.193	1.000^b^
2004 vs 2014	1.444	1.172 to 1.781	.024^b^
2009 vs 2014	1.341	1.062 to 1.693	.031^b^
5-year impact factor^c^			
2004 vs 2009	−.264	−1.659 to .629	.480
2004 vs 2014	.745	−.528 to 1.399	.219
2009 vs 2014	1.100	.328 to 1.842	.002
Assessment of reduced competence^a^			
2004 vs 2009	2.154	1.207 to 3.844	.020^b^
2004 vs 2014	1.579	1.018 to 2.448	.110^b^
2009 vs 2014	.733	.405 to 1.325	.289^d^
Potential conflicts of interest^a^			
2004 vs 2009	1.077	.737 to 1.573	1.000^b^
2004 vs 2014	3.333	1.748 to 6.358	.001^b^
2009 vs 2014	1.341	1.062 to 1.693	.031^d^
Number of ethics-related issues reported^e^			
2004 vs 2009	−0.96	−2.98 to 1.06	.494
2004 vs 2014	−2.33	−4.26 to −0.40	.014
2009 vs 2014	−1.37	−2.86 to 0.12	.078

*CI* Confidence Interval

^a^ Relative risk for the absence of the characteristic

^b^ Fisher exact test

^c^ Mann-Whitney test and Hodges-Lehmann estimator, data are median differences

^d^ Pearson χ^2^ test

^e^ Tukey’s test, data are mean differences

Median 5-year impact factor was 2.784 (1th quartile 2.130, 3rd quartile 4.626). Five-year impact factor was greater in 2009 than in the other two years, with no clear linear-by-linear change; post-hoc comparison indicated that it was significantly greater in RCTs published in the year 2009 compared to 2014 (Table 2). PEDro score ranged from 3 to 8 (median 6); there were no significant differences between years of publication (Table 1).

Neuromuscolar interventions were most frequently applied (53 studies), followed by musculoskeletal interventions (20 studies). Standard care was frequently used as side intervention, or comparator (32 studies). The outcome measures used to investigate the efficacy of the interventions mainly belonged to muscle and movement functions (21 studies), gait pattern functions (17 studies), balance (13 studies), walking (9 studies), arm-hand activities (26 studies), and activities of daily living (24 studies) of the International Classification of Functioning domains.

### Ethics reporting

**Fig. 3:**
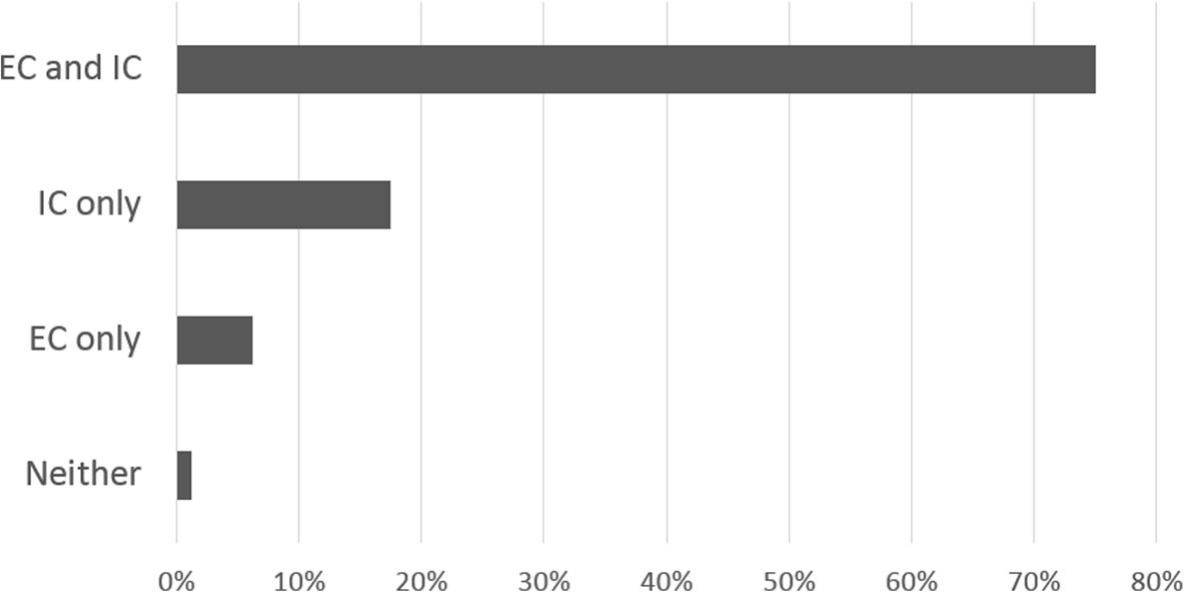
Ethic Committee approval and Informed Consent reporting. / Data are presented as percentages. EC = ethics review committee study approval. IC = obtainment of informed consent

Ethics committee or institutional review board study approval was reported in 65 (81.2%) studies, and 74 (92.5%) articles mentioned that consent was obtained. Details about the ethics committee and the consent process were available in half of the cases (51.2 and 52.5%, respectively) (Table 3). One paper failed to report both ethics committee approval and obtainment of informed consent (Fig. 3).

**Table 3:** Ethics-related issues reporting

Year	2004 (*n* = 13)	2009 (*n* = 28)	2014 (*n* = 39)	p	All studies (*n* = 80)
Ethic committee study approval	9 (69.2)	23 (82.1)	33 (84.6)	.470^b^	65 (81.2)
Details about ethic committee	6 (46.2)	13 (46.4)	22 (56.4)	.667^c^	41 (51.2)
Consent	11 (84.6)	26 (92.9)	37 (94.9)	.443^b^	74 (92.5)
Details about the consent process	4 (30.8)	16 (57.1)	22 (56.4)	.245^b^	42 (52.5)
Assessment of reduced cognitive competence	3 (23.1)	18 (64.3)	20 (51.3)	.049^b^	41 (51.2)
Incentives or compensation, and details	0 (0.0)	0 (0.0)	3 (7.7)	.278^b^	3 (3.8)
Funders and details	10 (76.9)	20 (71.4)	26 (66.7)	.848^b^	56 (70.0)
Potential conflicts of interest	3 (23.1)	8 (28.6)	30 (76.9)	<.001^b^	41 (51.2)
Statement about sample size estimates	5 (38.5)	8 (28.6)	17 (43.6)	.455^c^	30 (37.5)
Performing of power calculations	5 (38.5)	7 (25.0)	15 (38.5)	.478^c^	27 (33.8)
Appropriateness of comparators	10 (76.9)	24 (85.7)	34 (87.2)	.638^b^	68 (85.0)
Matching of comparators	11 (84.6)	19 (67.9)	30 (76.9)	.509^b^	60 (75.0)
Potential harm for participants	1 (7.7)	6 (21.4)	10 (25.6)	.470^b^	17 (21.2)
Reporting presence/absence of adverse events	4 (30.8)	7 (25.0)	20 (51.3)	.081^b^	31 (38.8)
Accordance with the Helsinki declaration.	0 (0.0)	3 (10.7)	6 (15.4)	.403^b^	9 (11.2)
Number of ethics-related issues reported^a^	7.5 (1.6)	8.5 (2.3)	9.9 (2.9)	.009^d^	9.0 (2.6)

Data are presented as absolute frequencies (percentages) except ^a^ mean (standard deviation)

^b^ Fisher exact test, ^c^ Pearson χ^2^ test, ^d^ one-way ANOVA

The Mini-Mental State Examination or similar test aimed to assess cognitive capacity were administered in the inclusion stage in 41 (51.2%) publications, namely in 3 (23.1%), 18 (64.3%), and 20 (51.3%) studies in year 2004, 2009, and 2014, respectively; this difference across the three years was borderline significant but had no linear-by-linear association (Table 3). In a post-hoc test a significantly greater probability that such assessment would not be performed was observed for studies published in the year 2004 compared to 2009 (Table 2), Only 3 papers published in 2009 specified how consent was acquired for vulnerable participants. Separate consents for videotaping or for publication of participant’s photos were reported, each in one study.

Incentives or compensation for participants (money or gifts, or free transportation to the research center) were stated in 3 articles published in 2014. Funders of the studies and details related were reported in 56 (70.0%) cases. The proportion of RCTs that declared the presence/absence of conflict of interest increased significantly across the three years, from 23.1% (3/13) in 2004, to 28.6% (8/28) in 2009, to 76.9% (30/39) in 2014 (Table 3). Authors declared conflict of interest only in 2 (2.5%) studies. Post-hoc comparisons showed that the probability that potential conflict of interest and related details were not reported was greater for trials published in the years 2004 and 2009 compared to those published in the year 2014 (Table 2).

Statements about sample size estimation were identified in 30 (37.5%) articles, 27 of which performed a power calculation. We considered appropriate the comparator interventions in 68 (85.0%) cases; participants received the same amount of attention in 60 (75.0%) trials, whereas in the remaining ones the attention was minor, or the control inactive. Justification for the use of placebo was always reported. Out of the 16 RCTs reporting the use of placebo/sham controls, the experimental intervention was represented by electrical stimulation in 10, by acupuncture in 2, by a combination of acupuncture and electrical stimulation in 2, by action observation training in 1, and by respiratory muscles training in 1.

Potential harm for participants was mentioned in 17 (21.2%) studies, and mainly included increased pain and/or fatigue, risk of falls, and unstable cardiovascular status. Plans for collecting, assessing, reporting, and managing adverse events and other unintended effects of trial interventions were reported respectively in 13, 11, 3, and 8 of these articles. The reporting of presence/absence of harm or adverse events was observed in 31 (38.8%) cases (Table 3). None of the studies mentioned eventual follow up care.

Although we found a clear reference to steps taken to protect anonymity in only one article, confidentiality was always preserved. Eighteen publications (30%) showed pictures depicting models or participants: with only two exceptions, faces were shown only in part (twice masking the eyes region) or were shielded. As previously noted, one study reported a specific consent for publication of the participant’s photos.

The number of ethics-related issues reported was significantly different across the three years considered (one-way ANOVA F(5.07) = 6.345, *p* = .009) (Table 3); Tukey post-hoc comparisons showed that this number was significantly higher in RCTs published in the year 2014 compared to 2004, whereas there was no significant difference between the years 2004 and 2009, and the years 2009 and 2014 RCTs (Table 2).

### Multivariable predictors of ethics reporting

Multiple regression showed that the number of ethics-related issues reported could be predicted as 1.167 + .893* year of publication (coded as 1 = 2004, 2 = 2009, and 3 = 2014) + .799* PEDro score (F_[4,75]_ = 11.103, *p* < .001, adjusted R^2^ of .338). Five-year impact factor and clinical trial registration (coded as 0 = absent and 1 = present) were not significant predictors (Table 4).

**Table 4:** Multiple regression analysis results

	B ± Std. Error	95% CI for B	β	t	*p*
(Costant)	1.167 ± 1.410	−1.641 to 3.975		.828	.410
Year of publication	.893 ± .368	.159 to 1.627	.251	2.424	.018
5-year Impact Factor	.261 ± .164	−.066 to .589	.162	1.591	.116
Clinical trial registration	.708 ± .721	−.727 to 2.144	.102	.983	.329
PEDro score	.799 ± .212	.376 to 1.222	.391	3.761	<.001

*B* regression coefficients, followed by the respective standard error, *CI* confidence interval; *β* standardized regression coefficient

## Discussion

In our study, we observed a broad variability in the reporting proportions of ethics-related components of research methods. Ethics committee approval and obtaining an informed consent, the main ethical issues, were the most reported. Approximately half of the studies reported details such as name and location of the committee, or how consent was acquired, but only 3 papers specified how consent was obtained for vulnerable participants. Information about funding was frequently reported (70% of studies), whereas that about conflict of interest was mentioned in about half of the studies, with a significantly increasing trend over the years. Statement about sample size estimates, potential harm for participants, and presence/absence of adverse events were definitively underreported, and details about incentives or compensation for participants and steps taken to protect confidentiality were almost ignored. The year of publication and PEDro score were associated with the completeness of the reporting of ethics-related issues.

### Limitations

Articles analyzed in the present study were limited in terms of year of publication (2004, 2009, and 2014). In the absence of references, we established a 20 % of the gained studies as the target sample. However, we made our calculation based on the total number of articles present in the PubMed-generated lists, and before the screening process. Thus, the representativeness of our random sample may be hypothesized higher than we expected. We performed our search on PubMed, which has been recognized as a comprehensive database indexing RCTs of physical therapy interventions [33]. To be indexed in PubMed, journals should demonstrate one certain quality of the editorial work, including features such as statements indicating adherence to ethical guidelines and evidence that authors have disclosed financial conflicts of interest. This condition suggests that in our sample the reporting of ethics issues may be of better quality than in the general population. Moreover, since we reviewed only English-language publications, we do not know if similar ethics reporting characteristics could be observed in non-English language publications. These factors limit the generalizability of the findings.

A wide range of ethical issues was considered in our study. When we drafted the protocol, we tried to be comprehensive. However, establishing which ethics-related issues should be contained in a fully comprehensive list would require consensus from a broad multidisciplinary expert team, which was beyond our intentions and capabilities. Extracting data on the reporting of the ethical aspects contained in our list was challenging in the absence of clear statements; for example, we found it difficult to extrapolate data on issues such as those related to the potential harm for participants. Thus, we acknowledge that objectivity might have not been maintained in these circumstances.

### Interpretation

Associations between methodological quality and ethics reporting practices have already been observed, [3] as well as improvement of ethical approval and consent reporting in RCTs [34]. Compared to a previous study on physical therapy publications, we observed a greater rate of trials reporting both ethics committee approval and consent obtainment (+ 11%), as well as a smaller rate of those who did not report either (− 16%) [14].

The reporting of research ethics-related issues and methods observed in our sample met, to some extent, the International Committee of Medical Journal Editors recommendations [13]. The statement underlines that details about the approval from an ethics committee, the obtaining of informed consent, and the research funding should be reported while publishing an article. The presence of conflict of interest is also highly emphasized, and we notice in our sample a significant increase over time in the related reporting. The document does not clearly formulate recommendations about the ethical issues that we found not properly reported. Nevertheless, ethics aspects like sample size dimension and risk of harm for participants are relevant [10, 18, 35, 36]. Compensation to participants may impact the statistical inferences, and transparency on the topic is desirable when reporting clinical trials [37].

Despite the progress observed, and in accordance with other authors, [35] our findings suggest that the reporting of many ethics-aspects needs to be improved in RCTs of physical therapy interventions after stroke. Reporting guidelines should be updated [35]; however, adding brief and clear sentences to the text (e.g., “The occurrence of adverse events has been monitored”, “No compensation was offered to participants”, “Data were managed and accessed only by authorized personnel”) could be a starting point.

## Conclusion

Though improved over time, ethics reporting practices in RCTs of physical therapy interventions after stroke should be ameliorated. With its limitations, our study shows deficiencies of various degrees. Authors, editors, and reviewers should be more rigorous and demanding about the reporting of ethic-related methods regarding the reproducibility of research. Almost all the ethics-related issues in our list have been recognized as part of the minimum set of items to be addressed drawing a protocol for a RCT [28]. If protocols are drawn up in accordance with the expected standard, and subsequent trials are reported faithfully, this should result in an increase in quantity and quality of the ethics reporting practices in RCTs. Thus, the understanding of ethical methods and the convergence on best practices will be promoted, [12, 28] and a virtuous circle originated.

## Appendix

**Table 5:** Questions

^a^1.0	Was ethics review committee or institutional review board study approval reported?	^a^6.0	Were information about sample size estimates reported?
1.1	Was the name of the committee reported?	^a^6.1	Were power calculations performed?
1.2	Was the location of the committee reported?	^a^7.0	Were comparator interventions appropriate?
^a^1.4	Were details about the ethics committee reported?	^a^7.1	Was the dosage of the intervention provided to groups comparable?
^a^2.0	Was it reported if informed consent was obtained?	8.0	Was the eventual use of placebo justified?
2.1	Was the consent for participation written?	^a^9.0	Was potential harm for participants mentioned?
2.2	Was the consent for participation oral?	^a^9.1	Were plans for collecting adverse events and other unintended effects mentioned?
2.3	How was consent (and possibly assent) acquired for members of vulnerable populations?	^a^9.2	Were plans for assessing adverse events and other unintended effects mentioned?
^a^2.4	Were details about the consent process reported?	^a^9.3	Were plans for reporting adverse events and other unintended effects mentioned?
^a^3.0	Were incentives or compensation for participants reported?	^a^9.4	Were plans for managing adverse events and other unintended effects mentioned?
3.1	What did they receive, money, a gift, free medical care or treatment, free transportation or other services?	^a^9.5	Were presence/absence of harm or adverse events reported?
3.2	Were details about incentives or compensation given to participants reported?	^a^10.0	Was mentioned if appropriate follow up care was assured?
^a^4.0	Were funders reported?	^a^11.0	Were steps taken to assess if participants had reduced competence (eg. use of validated screening tools for cognitive ability) reported?
4.1	How many organizations were involved?	11.1	Which measures were taken to protect participants with reduced competence best interests?
4.2	What were the funding sources? Governmental agencies, private foundations, or some other type?	^a^12.0	Were steps taken to prevent unauthorized access to personal and clinical data (confidentiality) mentioned?
4.3	Were details about funders reported?		
^a^5.0	Were there statements about potential conflicts of interest?	^a^12.1	Was confidentiality preserved?
5.1	Was any conflict declared?	^a^13.0	Was reported if the study was conducted in accordance with the Helsinki Declaration?
5.2	Were details about conflicts of interest reported?		

^a^item counted in the number of ethics-related issues reported

## Abbreviation

RCT: Randomized controlled trial

## References

[1] Vergnes JN, Marchal-Sixou C, Nabet C (2010). Ethics in systematic reviews. J Med Ethics.

[2] Jacobsen KH (2009). Reporting of ethics-related methods in epidemiological research. J Med Ethics.

[3] Ruiz-Canela M, de Irala-Estevez J, Martínez-González MA (2001). Methodological quality and reporting of ethical requirements in clinical trials. J Med Ethics.

[4] Ashton CM, Wray NP, Jarman AF, Kolman JM, Wenner DM, Brody BA (2011). A taxonomy of multinational ethical and methodological standards for clinical trials of therapeutic interventions. J Med Ethics.

[5] Roberts LW (1999). Ethical dimensions of psychiatric research: a constructive, criterion-based approach to protocol preparation. The research protocol ethics assessment tool (RePEAT). Biol Psychiatry.

[6] Harriss DJ, Atkinson G (2015). Ethical standards in sport and exercise science research: 2016 update. Int J Sports Med.

[7] Chiumento A, Rahman A, Frith L, Snider L, Tol WA (2017). Ethical standards for mental health and psychosocial support research in emergencies: review of literature and current debates. Glob Health.

[8] Junod V, Elger B (2010). Retrospective research: what are the ethical and legal requirements?. Swiss Med Wkly.

[9] Emanuel EJ, Wendler D, Grady C (2000). What makes clinical research ethical?. JAMA.

[10] Sim J (1989). Methodology and morality in physiotherapy research. Physiotherapy.

[11] Maher CG, Sherrington C, Elkins M (2004). Challenges for evidence-based physical therapy: accessing and interpreting high-quality evidence on therapy. Phys Ther.

[12] Anderson JA, Eijkholt M, Illes J (2013). Ethical reproducibility: towards transparent reporting in biomedical research. Nat Methods.

[13] Recommendations for the conduct, reporting, editing, and publication of scholarly work in medical journals [Internet]. ICMJE, The International Committee of Medical Journal Editors [accessed 2016 Dec 10]. Available from: http://www.icmje.org/icmje-recommendations.pdf 25558501

[14] Henley LD, Frank DM (2006). Reporting ethical protections in physical therapy research. Phys Ther.

[15] Goodman SN, Fanelli D, Ioannidis JP (2016). What does research reproducibility mean?. Sci Transl Med.

[16] Eijkholt M, Anderson JA, Illes J (2012). Picturing neuroscience research through a human rights lens: imaging first-episode schizophrenic treatment-naive individuals. Int J Law Psychiatry.

[17] Weingarten MA, Paul M, Leibovici L (2004). Assessing ethics of trials in systematic reviews. BMJ.

[18] Sim J (2010). Addressing conflicts in research ethics: consent and risk of harm. Physiother Res Int.

[19] Sabapathy SS, Janakiraman K, Swarnalatha CC (2010). Reporting of ethical issues in indian physiotherapy journals. J Phys Ther.

[20] Moral-Munoz JA, Arroyo-Morales M, Herrera-Viedma E, Cobo MJ. An overview of thematic evolution of physical therapy research area from 1951 to 2013. Front Res Metr Anal 2018;3: article 13. doi:10.3389/frma.2018.00013

[21] von Elm E, Altman DG, Egger M (2008). STROBE initiative. The strengthening the reporting of observational studies in epidemiology (STROBE) statement: guidelines for reporting observational studies. J Clin Epidemiol.

[22] DeJong G, Horn SA, Gassaway JA (2004). Toward a taxonomy of rehabilitation interventions: using an inductive approach to examine the ‘black box’ of rehabilitation. Arch Phys Med Rehabil.

[23] American Physical Theraphy Association (2001). Guide to physical theraphy practice.

[24] Clinician’s Handbook, Motor rehabilitation [internet]. Evidence-Based Review of Stroke Rehabilitation. Available from: http://www.ebrsr.com/clinician-handbook. [Accessed 2017 Jan 22]

[25] Pinto RZ, Elkins MR, Moseley AM (2013). Many randomized trials of physical therapy interventions are not adequately registered: a survey of 200 published trials. Phys Ther.

[26] Urbaniak GC, Plous S (2015). Research Randomizer (Version 4.0) [Computer software]. Retrieved on January 22, 2017, from: http://www.randomizer.org/

[27] 2014 Journal Citation Reports ® Science Edition (Clarivate Analytics, 2017). Available from: https://jcr.incites.thomsonreuters.com/JCRJournalHomeAction.action. [accessed 2017 Jan 5]

[28] Chan AW, Tetzlaff JM, Altman DG (2013). SPIRIT 2013 statement: defining standard protocol items for clinical trials. Ann Intern Med.

[29] Caprino L, Russo P (2006). Developing a paradigm of drug innovation: an evaluation algorithm. Drug Discov Today.

[30] Armijo Olivo S, Macedo LG, Gadotti IC (2008). Scales to assess the quality of randomized controlled trials: a systematic review. Phys Ther.

[31] Maher CG, Sherrington C, Herbert RD (2003). Reliability of the PEDro scale for rating quality of randomized controlled trials. Phys Ther.

[32] Foley NC, Teasell RW, Bhogal SK, Speechley MR (2003). Stroke rehabilitation evidence-based review: methodology. Top Stroke Rehabil.

[33] Michaleff ZA, Costa LO, Moseley AM (2011). CENTRAL, PEDro, PubMed, and EMBASE are the most comprehensive database indexing randomized controlled trials of physical therapy interventions. Phys Ther.

[34] Schroter S, Plowman R, Hutchings A (2006). Reporting ethics committee approval and patient consent by study design in five general medical journals. J Med Ethics.

[35] Trung LQ, Morra ME, Truong ND (2017). A systematic review finds underreporting of ethics approval, informed consent, and incentives in clinical trials. J Clin Epidemiol.

[36] Cesana BM, Antonelli P (2016). Sample size calculations in clinical research should also be based on ethical principles. Trials.

[37] Swanson DM, Betensky RA (2015). Research participant compensation: a matter of statistical inference as well as ethics. Contemp Clin Trials.

